# Systematic investigation and microbial community profile of indole degradation processes in two aerobic activated sludge systems

**DOI:** 10.1038/srep17674

**Published:** 2015-12-11

**Authors:** Qiao Ma, Yuanyuan Qu, Xuwang Zhang, Ziyan Liu, Huijie Li, Zhaojing Zhang, Jingwei Wang, Wenli Shen, Jiti Zhou

**Affiliations:** 1 Key Laboratory of Industrial Ecology and Environmental Engineering (Ministry of Education), School of Environmental Science and Technology, Dalian University of Technology, Dalian 116024, People’s Republic of China

## Abstract

Indole is widely spread in various environmental matrices. Indole degradation by bacteria has been reported previously, whereas its degradation processes driven by aerobic microbial community were as-yet unexplored. Herein, eight sequencing batch bioreactors fed with municipal and coking activated sludges were constructed for aerobic treatment of indole. The whole operation processes contained three stages, i.e. stage I, glucose and indole as carbon sources; stage II, indole as carbon source; and stage III, indole as carbon and nitrogen source. Indole could be completely removed in both systems. Illumina sequencing revealed that alpha diversity was reduced after indole treatment and microbial communities were significantly distinct among the three stages. At genus level, *Azorcus* and *Thauera* were dominant species in stage I in both systems, while *Alcaligenes*, *Comamonas* and *Pseudomonas* were the core genera in stage II and III in municipal sludge system, *Alcaligenes* and *Burkholderia* in coking sludge system. In addition, four strains belonged to genera *Comamonas*, *Burkholderia* and *Xenophilus* were isolated using indole as sole carbon source. *Burkholderia* sp. IDO3 could remove 100 mg/L indole completely within 14 h, the highest degradation rate to date. These findings provide novel information and enrich our understanding of indole aerobic degradation processes.

Research of indole has been drawing increasing concerns recently. It not only acts as the intercellular signal molecule in bacterial systems, but also a plant-growth modulator as well as an interkingdom signal between microbiota and mammalian hosts^1^,^2^,^3^,^4^. Indole plays diverse signaling roles in controlling virulence, biofilm formation, plasmid stability and stress responses, thus affecting the phenotypes and physiological behaviors of microbes^1^,^5^. Studies have shown that indole can directly and rapidly cross bacterial membrane without Mtr, AcrEF-TolC or other transporter proteins, facilitating its microbial and interkingdom signaling functions^6^. It has been proven that over 85 bacterial species can produce indole from tryptophan due to the existence of tryptophanase^1^. Furthermore, indole is a genotoxic and recalcitrant *N*-heterocyclic aromatic pollutant which can be generated in large quantity from coal mining and oil shale operations^7^,^8^. Therefore, microbes will inevitably encounter and interact with indole in various matrices, such as wastewater.

Bacteria can produce indole from tryptophan and also harbor the ability to degrade indole. For this regard, initial attempts could be dated back to 1920s, when Raistrick and Clark found that *Bacillus* spp. could attack indole nucleus and release ammonia^9^. Afterwards, Sakamoto *et al.*, Fujioka and Wada, and Claus and Kutzner further enriched indole degradation pathways using different aerobic bacterial strains^10^,^11^,^12^. In recent years, *Pseudomonas* and *Cupriavidus* spp. have also been isolated and characterized for indole degradation^13^,^14^,^15^,^16^,^17^. Under aerobic conditions, indole degradation was initiated via hydroxylation at C3 or C2/C3 positions resulting in indoxyl or 2,3-dihydroxyindole formation. Anthranilic acid was the typical intermediate and indigoids were also frequently formed^10^,^11^,^12^,^15^. Under anaerobic conditions, indole was usually attacked at C2 position with 2-oxindole generation under denitrifying, methanogenic and sulfate-reducing conditions^18^,^19^,^20^. Denitrifying microbial communities could effectively convert the intermediate 2-oxindole leading to a higher indole degradation efficiency^20^. It is interesting that very limited anaerobic pure microorganisms have been isolated and characterized^21^. The structures of bacterial communities under denitrifying and sulfate-reducing conditions have been identified using 16S rRNA gene clone libraries^22^. However, the bacterial community for indole aerobic degradation, which existed ubiquitously, has not been referred yet to the best of our knowledge. With the ongoing development of sequencing technology, it is possible to explore the entire profile of microbial community previously unattainable using high-throughput sequencing technologies. Especially, Illumina sequencing has been widely used due to its low cost and high sequencing depth merits^23^. Therefore, application of Illumina sequencing will provide huge amounts of useful information to enhance our understanding of indole degradation microbial communities.

Investigations on wastewater treatment plant (WWTP) sludges revealed that core populations existed in either municipal activated sludge or certain type of industrial sludge despite of different geographical locations^24^,^25^. Indeed, the microbial community composition and structure are resulted from various factors classified as deterministic and stochastic processes^26^. Deterministic factors play key roles in community assembly of wastewater treatment systems. Among them, original microbial community structures play a role in community formation processes due to the different interspecies interactions (e.g., commensalism, competition, predation, synergism and tradeoffs) and species traits. From this point, two different types of original sludges from the secondary sedimentation tanks of a local municipal WWTP and a coking WWTP were used in the present study. Since microbes could utilize indole as the sole carbon source or co-metabolize indole in the presence of other carbon sources, three different cases were investigated using sequencing batch reactor systems: (i) co-metabolism with glucose and nitrogen supplementation, (ii) indole as the sole carbon source with nitrogen supplementation and (iii) indole as the sole carbon and nitrogen source. Meanwhile, isolation of pure cultures in each operation stage was also conducted to obtain available indole-degrading resources, which could be a supplement for the metagenomic sequencing. The main goal of present study was to comprehensively and systematically reveal indole aerobic degradation microbial communities, enriching our understanding of indole-degrading processes.

## Results

### Performance of the indole-degrading bioreactors

**Figure 1:**
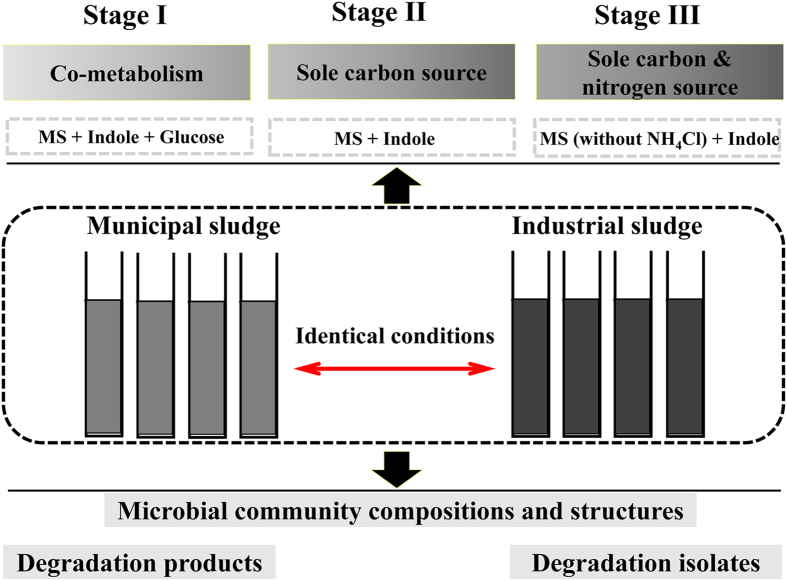
Experimental design of indole aerobic degrading processes. Two kinds of activated sludge were adopted and each sludge was operated in quadruplicate. The whole operation processes contained three stages and each stage lasted for two months. Stage I, indole and glucose were the carbon source and NH_4_Cl was the nitrogen source. Stage II, indole was the sole carbon source and NH_4_Cl was the nitrogen source. Stage III, indole was the sole carbon and nitrogen source. The influent indole concentration was 200 mg/L throughout the experiment. MS medium stands for mineral salt medium.

The construction and operation processes of the reactors were shown in Fig. 1. Two types of activated sludges, i.e. municipal sludge (system A) and industrial sludge (system B), were gathered and operated in quadruplicate. Overall, indole and chemical oxygen demand were removed completely within every operation cycle. The sludge samples at each final stage were centrifuged, and the pellets were dissolved in dimethyl sulfoxide. Apparently, the product colors differed between the two sludge systems (Figure S1). System A displayed dark brown while system B was blue, similar with our previous study^27^. The products were further analyzed using high performance liquid chromatography**-**mass spectrometry (HPLC-MS) and HPLC analyses (Figure S2). Indigo (retention time 17.9 min) with m/z of 261.067 (M-H^–^) was the major product in both systems at any stage. Indirubin (retention time 22.5 min), an isomer of indigo, was also formed although in low production. It was found that at least three substances with m/z 261.067 (M-H^–^) were detected in both systems at any stage, indicating that some other indigoids were also produced besides indigo and indirubin. These findings indicated that the distinct colors of system A and B should be resulted from different proportions of these products.

### Overview of the 16S rRNA high-throughput sequencing results

After sequence treatment, 10,833–56,298 clean reads were obtained for each sample which were then set a resample at 10,000 for following analysis. Richness and alpha diversity of the microbial community indicated by Chao1 value, OTU number, Shannon and Simpson indexes were calculated as shown in Table 1. For system A, the total OTU numbers and Chao1 values of the original sludge, 629 ± 8 and 800 ± 56, were much higher than those of other stages. Shannon index and Simpson index data demonstrated that indole could significantly reduce sludge diversity with the diversity order of original sludge > Stage I > Stage II > Stage III, the same as the OTU and Chao1 numbers. As the case of system B, a similar trend was exhibited with system A except that the diversity in stage I was higher than original one. Overall, richness and diversity of both systems in stage II and stage III were very low with Chao1 183–324, OTU 108–188, Shannon index 1.73–2.54 and Simpson index 0.16–0.31.

**Figure 2:**
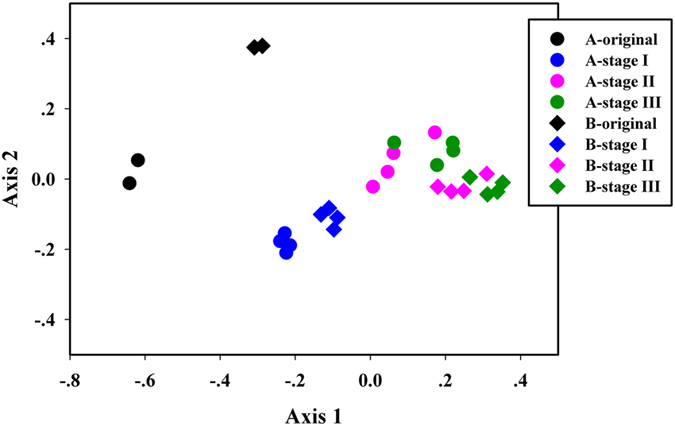
Non-metric multi-dimensional scaling ordination (NMDS) plot of all sludge samples. The further distance between the points, the higher dissimilarity it is. Data is OTU-level. This figure shows a time-dependent clustering pattern.

The non-metric dimensional scaling (NMDS) ordination plot which depicted microbial community differences at OTU level was shown in Fig. 2. Microbial communities with indole dosing were clearly different from that of original sludge, and the communities changed with operation stages from left to right in Axis 1 direction. At stage I, microbial communities of A and B were clearly separated. The points for stage II and stage III were mixed with each other, implying that microbial communities at these stages might be similar to a certain degree. Heat map plot at OTU level (Figure S3) showed that microbial community clearly shifted from the original ones. Meanwhile, the quadruplicate samples of any group clustered together, suggesting the results were credible. Three significance tests, i.e. MRPP, ANOSIM and Adonis, were conducted to analyze the differences of sludge communities at each stage (Table 2). *P* value less than 0.05 meant a significant difference between the communities. Although microbial communities of system A and B exhibited similar variation tendency according to NMDS plot, dissimilarity analyses declared that sludge communities were significantly distinct from each other of each stage (All *P* < 0.05). The communities of system A and B at same stage were also notably different.

### Bacterial communities of original sludge

**Figure 3:**
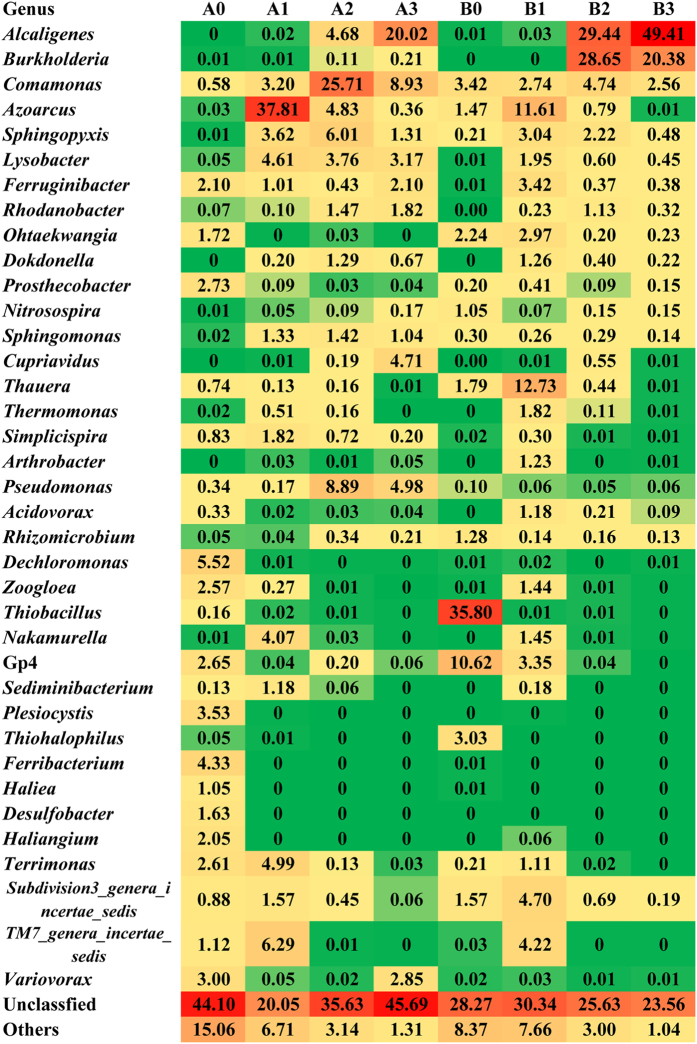
Major genera of all groups. Major means sequence percentage is above 1% in any group. A0, A1, A2, A3 stand for the samples of original, stage I, stage II and stage III of system A. B0, B1, B2, B3 stand for the samples of original, stage I, stage II and stage III of system B. The redder the color, the higher the percentages, and the bluer the color, the lower the percentages.

The microbial compositions and structures of the two sludge systems at phylum, class and genus level were analyzed in detail (Fig. 3, Figure S4). Obviously, the original community of system A and B were quite different. *Proteobacteria* (51.66%) and *Bacteroidetes* (24.64%) were the dominating phyla in system A, while *Proteobacteria* (71.96%) and *Acidobacteria* (11.08%) dominated in system B. Typical genera in municipal sludge, such as *Zoogloea* (2.57%), *Dechloromonas/Ferribacterium* (9.85%), Gp4 (2.65%) and *Prosthecobacter* (2.73%), were detected as the main components in system A as well.Genus *Thiobacillus* (35.80%) was the foremost component in system B, which was responsible for denitrification and thiocyanate biodegradation processes ubiquitously appeared in coking WWTPs. This result was consistent with previous studies on WWTP sludges using high-throughput sequencing^24^,^25^.

### Bacterial communities using glucose-indole as co-substrate

After a course of two-month operation feeding glucose-indole and NH_4_Cl as the carbon and nitrogen sources, both sludge communities significantly shifted. *Proteobacteria*, *Bacteroidetes* and *Acidobacteria* were the major phyla in both sludges. At genus level, *Azoarcus* was the primary component in system A, accounting for 37.81% of the total sequences. Other major genera included *TM7_genera_incertae_sedis* (6.29%), *Terrimonas* (4.99%), *Nakamurella* (4.07%), *Lysobacter* (4.61%), *Sphigopyxis* (3.62%) and *Comamonas* (3.20%). Genera such as *Zoogloea*, *Dechloromonas/Ferribacterium*, Gp4 and *Prosthecobacter* which were dominating in original sludge were absent in this stage. In system B, *Thauera* and *Azoarcus* were the two primary genera with sequence percentages of 12.73% and 11.61%, respectively. *Thiobacillus* decreased dramatically from 35.80% (original sludge) to 0.01% (stage I). *Alcaligenes* and *Burkholderia* were hardly detected in both systems in stage I, and *Comamonas* was in a modest proportion with percentage of 3.31%.

### Bacterial communities using indole as the sole carbon source

After continuous dosing of indole and NH_4_Cl as the carbon and nitrogen sources for another two months, bacterial community experienced a great change in both systems compared with those of stage I. The two systems harbored similar components at high taxonomic level. Both systems contained four major classes, i.e. *Betaproteobacteria*, *Gammaproteobacteria*, *Alphaproteobacteria* and *Sphingobacteria*, among which *Betaproteobacteria* and *Gammaproteobacteria* were extraordinary dominant (over 87%). However, community structures differed from each other at low taxonomic level. *Comamonas* increased to be the foremost genus in system A, accounting for 25.71% of the total sequences, followed by *Pseudomonas* (8.89%), *Sphingopyxis* (6.01%), *Azoarcus* (4.83%), *Pusillimonas* (4.68%), *Lysobacter* (3.76%), *Rhodanobacter* (1.47%), *Sphingomonas* (1.42%) and *Dokdonella* (1.29%). It was worth noting that *Azoarcus* decreased from 37.81% to 4.83%. For system B, the two hardly detected genera in stage I, *Alcaligenes* and *Burkholderia*, became the core genera accounting for 29.44% and 28.65%, respectively. Other major genera contained *Comamonas* (4.74%), *Sphingopyxis* (2.22%) and *Rhodanobacter* (1.13%). *Thauera*, the predominate genus in stage I (12.73%), occupied only 0.44% in this stage.

### Bacterial community using indole as the sole carbon and nitrogen source

At the final stage, bacterial composition and structure were similar with those of stage II at phylum and class levels in both systems. *Alcaligenes* increased continuously reaching 20.02% and 49.41% in system A and B, respectively, while *Azozrcus* decreased continuously to be the minor genus in both systems (0.36% in A and 0.01% in B). *Comamonas* also decreased to some extent with percentages of 8.93% and 2.56% in sludge A and B, respectively. *Burkholderia* was still scarce in system A (0.21%) but dominating in system B (20.38%). Other major genera in system A contained *Pseudomonas* (4.98%), *Cupriavidus* (4.71%), *Lysobacter* (3.17%), *Variovorax* (2.85%), *Ferruginibacter* (2.10%), *Rhodanbacter* (1.82%), *Sphingopyxis* (1.31%) and *Sphingonmonas* (1.04%). *Cupriavidus* was increasing as the major genus for the first time in system A, but absent in system B. *Pseudomonas* was still acting as a major component in system A which was also sporadic in system B. In system B, *Alcaligens*, *Burkholderia* and *Comamonas* were the only three major genera.

### Degradation of indole by isolated pure cultures from the reactors

**Figure 4:**
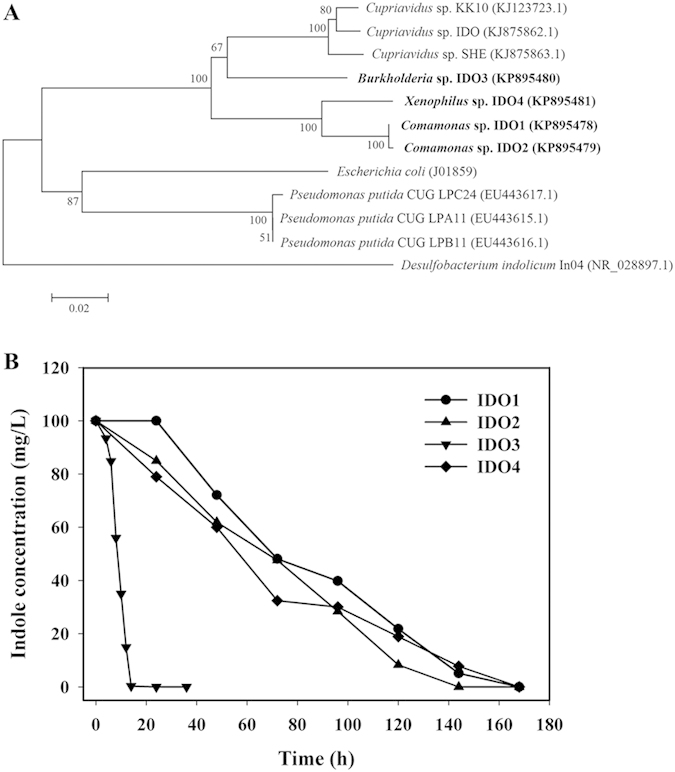
Phylogenetic tree (a) and indole degradation curves (b) of the four isolated indole bacteria. Phylogenetic tree was constructed using neighbor-joining method and numbers indicated the bootstrap values derived from 1000 replicates. Except for *E. coli*, all strains are reported indole degrading bacteria. Sequences were aligned using Clustal X 1.8 and MEGA 5.0 was used to construct phylogenetic tree.

To further explore microbial resources for indole degradation, we have tried to isolate pure indole degraders from these systems. Totally four strains, designated as IDO1, IDO2, IDO3 and IDO4 were obtained. Nearly the complete 16S rRNA gene sequences were obtained and they were identified as *Comamonas* sp., *Comamonas* sp., *Burkholderia* sp., and *Xenophilus* sp., respectively. According to the 16S rRNA gene sequences, a phylogenetic tree was constructed with the sequences of other indole degrading bacteria (Fig. 4A). *Comamonas* could be isolated at each stage of the operation, *Burkholderia* was obtained at stage II and stage III, and *Xenophilus* was only isolated in stage III (Table S1). This was in accordance with the Illumina sequencing data that *Comamonas* was abundant throughout the stages, and *Burkholderia* was emerging at stage II. However, other reported indole degrading bacteria, such as *Alcaligenes* spp., *Pseudomonas* spp. and *Cupriavidus* spp., were not obtained, which might be due to the restriction of present isolation method.

The indole-degrading characteristics of the four strains were investigated. All four strains could use indole as the sole carbon and nitrogen source, although the isolation medium contained NH_4_Cl as the nitrogen source. *Commonas* sp. IDO1 and IDO2 could transform indole and yield yellow substances, but bacterial density was quite low with hardly increased OD_600_ values. *Xenophilus* sp. IDO4 also produced yellowish products. The degradation efficiencies of these three strains were not comparable and almost one week was required to remove 100 mg/L indole. *Burkholderia* sp. IDO3 could utilize indole efficiently and remove 100 mg/L indole within 14 h, whose efficiency was the highest to date (Fig. 4B).

## Discussion

Concerns on indole have been exponentially increasing in recent years for its important roles in pharmaceutical industry and signaling functions^1^,^3^,^28^. However, indole transformation is actually an unrevealed and complicated process yet to be fully understood. For instance, many unidentified metabolites catalyzed by P450 hydroxylase and phenol hydroxylase were observed^29^,^30^, indole-degrading functional genes or gene clusters have never been acquired^11^,^31^, and indole aerobic degrading microbial communities have not been fully explored yet^27^. In this study, it was proven that indole could be effectively degraded in SBR activated sludge systems. Degradation efficiency (200 mg/L indole completely removed within two days) was much higher than that of anaerobic sludge systems^18^,^19^,^20^. HPLC-MS analysis verified that indigoids were produced and accumulated, which was in accordance with our previous study^27^. Production of indigo and its isomers suggested indole should be oxidized at C3 position leading to indoxyl generation, the precursor of indigo^32^. C3 position is more vulnerable and active than C2 thus easier attacked by oxygen catalyzed by aromatic oxygenases. Under anaerobic conditions, C2 was generally hydroxylated with 2-oxindole production which could be accumulated under methanogenic and sulfate-reducing but not under denitrifying conditions^20^,^33^. In both cases, the functional oxygenases for indole-degrading microbes have not been acquired to date.

Indole is toxic to organisms including microbes. It can cause membrane derangement, and high concentration of indole may inhibit energy production and protein folding by a transcriptomic and *in vitro* protein-refolding assay^34^,^35^. Meanwhile, studies have shown that indole is a proton ionophore which can arrest bacterial growth and cell division^36^. Therefore, indole as an exogenous stress would negatively affect some bacterial species. The diversity and richness of both system A and B decreased significantly, while it was not the case for stage I of system B whose diversity and richness were higher than original sludge. Sludge B was adopted from coking WWTP, where the wastewater contained large amount of inorganic and organic pollutants, such as phenol, naphthalene, indole, quinolone and pyridine^24^,^37^. In stage I, the influent was mineral salt medium, glucose and indole, which was less complicated than real coking wastewater. Hence, the influent might be less toxic than real coking wastewater leading to the higher alpha diversity and richness in reactor sludge.

**Figure 5:**
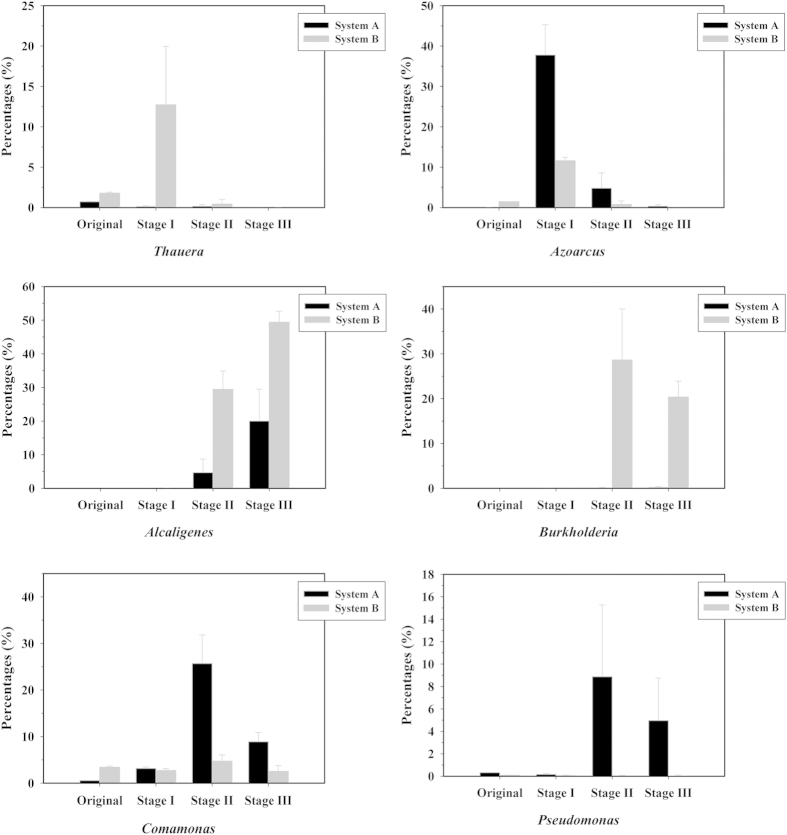
Proportions of the core genera for indole degradation in each stage. Data were calculated using the quadruplicates of each system.

Dominant species for indole degradation under the three investigated conditions were notably different, particularly for stage I with glucose and indole as the carbon sources (Fig. 5). *Azoarcus* (37.81%) was the most abundant genus in system A, and *Azoarcus* (11.61%) and *Thauera* (12.73%) were the top two genera in system B. *Thauera* and *Azoarus* were two functionally important and closely related species often occurred together responsible for denitrifying in activated sludge^38^. They also exhibited versatile degradation capacities towards a variety of aromatics^24^. Previous studies indicated that *Thauera* spp. could utilize indole as the sole carbon source under anaerobic conditions^39^,^40^. Hong *et al.* constructed a denitrifying reactor fed with indole and glucose as carbon sources, and *Thauera* was among the abundant genera revealed by 16S rRNA gene clone library analysis^22^. Afterwards, they isolated three *Thauera* spp. from a coking WWTP, all of which could degrade indole under aerobic conditions^41^. Taken together, these results suggested *Thauera* spp. were the common indole degraders in microbial community especially in the presence of other carbon sources. *Azoarus* was also identified as a frequent aromatic (e.g. quinoline) degrader in activate sludge^42^. Although reports on the degradation of indole by *Azoarus* were limited, the high proportion of this genus in stage I might be an evidence of its indole degradation potential.

The bacterial community compositions and core genera of stage II and III were similar to some extent. *Alcaligenes* was the key species in both sludge and increased to be the foremost genus in stage III. Indole degradation by *Alcaligens* has been documented previously and it was also dominant in the indole denitrifying sludge conducted by Hong *et al.*^12^,^22^. We have also conducted another bioaugmented indole degrading systems and found that *Alcaligenes* was increasing continuously and even reaching over 50% finally^43^. Thus *Alcaligenes* might be the functionally important indole degrading bacterial species in activated sludge systems. *Comamonas* was major in both systems throughout the operations with proportions 2.74–25.71%. Although it is an amazing aromatic degrading genus widespread in nature, it has never reported to be able to degrade indole yet^24^,^25^,^27^,^44^. *Pseudomonas*, the most studied species for indole degradation^13^,^14^,^45^, was dominant in system A (8.89% stage II and 4.98% stage III) but almost negligible in system B. On the contrary, *Burkholderia*, another well-known aromatic-degrading genus, was the main component in system B (28.65% stage II and 20.38% stage III) but negligible in system A. The research of *Burkholderia* on indole degradation has also been reported, which, however, could only co-metabize indole^46^. Since the operational and environmental conditions of these two systems were identical throughout the experiment, it could be concluded that original sludge composition and structure, accompanied by different interspecies interactions and species traits, would affect the community assemble processes. It was clearly shown that the microbial community composition and structure in stage II & III were significantly distinct from stage I. When indole was co-metabolized, *Thauera* and *Azoarus* were the major bacteria, while the typical aromatic degraders such as *Alcaligenes*, *Burkholderia* and *Pseudomonas* dominated the community when indole was utilized as sole carbon source. Understanding of indole degradation genetic mechanisms using metaproteomic and metatranscriptomic analyses is a valuable topic that has not been explored yet, and the corresponding indole oxygenases in the sludges should have a potential application in industrial field.

Four bacterial strains that could use indole as sole carbon and nitrogen source were isolated. 16S rRNA gene analysis indicated that they belonged to *Comamonas*, *Burkholderia* and *Xenophilus*, all of which have not been reported to assimilate indole as sole carbon source. *Comamonas* and *Burkholderia* were the major genera in the microbial communities, and their capacity for indole degradation further proved their functions in SBR systems. Through indole degradation curves, it was clearly shown that *Burkholderia* sp. IDO3 could degrade indole very efficiently. It could completely remove 100 mg/L indole within 14 h, the highest indole degradation rate to date^16^,^17^,^47^. *Alcaligenes* and *Pseudomonas*, two typical genera capable for indole degradation, were not obtained in the present study. The possible reason might be that the screening medium was not suitable for strain growth. Another genus, i.e. *Cupriavidus*, was also not obtained though its proportion in stage III of system A reached 4.71%. At the same time of present work, we also tried to isolate more indole degrading resources from other soil and sludge samples. Two strains named *Cupriavidus* sp. SHE and *Cupriavidus* sp. IDO were gained with superior indole degrading performance^16^,^17^. Indole degradation by these pure-culture strains displayed two different phenomena. Some strains transformed indole into yellowish products with limited strain growth (e.g., *Comamonas* sp. IDO1, *Comamonas* sp. IDO2 and *Cupriavidus* sp. SHE), while others could utilize indole more effectively and grow very well with production of indigoids (e.g., *Cupriavidus* sp. IDO and *Burkholderia* sp. IDO3). The different degradation performances might be due to the genetic differences of these strains. However, studies on indole degradation are just focused on strain isolation and metabolite identification, and attempts to identify the functional indole hydroxylase or dioxygenase have not been successful. To in-depth explore the underlying genetic mechanism of indole degradation, a combination of genomic and proteomic experiment was in progress.

In summary, culture-independent high throughput sequencing and culture-dependent methods were utilized to reveal the microbial composition and structure of two indole aerobic degradation sludge communities, i.e. municipal sludge system and coking sludge system, for the first time. *Azoarcus* and *Thauera* were the major genera when indole was co-metabolized with glucose as additional carbon source. When indole was added as the sole carbon source, *Alcaligenes, Comamonas* and *Pseudomonas* were the core species in municipal sludge system, while *Alcaligenes*, *Burkholderia* and *Comamonas* were the major genera in coking sludge systems. Four strains belonged to *Comamonas*, *Burkholderia* and *Xenophilus* were also isolated from the sludge systems and exhibited excellent efficiency for aerobic indole degradation, especially for *Burkholderia.*

## Materials and Methods

### Reactor design and operation

Municipal and industrial activated sludges were gathered from the secondary sedimentation tanks of Chunliu River WWTP (Dalian, China) and Benxi steel coking WWTP (Benxi, China). The sludges were stored at −4 °C immediately after sampling. Eight sequencing batch reactors (SBRs) with working volume of 1.0 L were constructed, among which four replicates were set for each kind of sludge. Mineral salt (MS) medium in the present study composed of (mg/L) 40 KH_2_PO_4_, 180 NH_4_Cl, 5 NaCl, 25 MgSO_4_·7H_2_O, 24 CaCl_2_, 20 FeSO_4_·7H_2_O. The SBRs were operated for six months, which were divided into three stages (2 months per stage). Stage I, influent was MS medium, 400 mg/L indole and 1000 mg/L glucose; stage II, influent was MS medium and 400 mg/L indole; stage III, influent was NH_4_Cl-free MS medium and 400 mg/L indole. Before operation, the sludges were acclimated for two weeks using stage I influent. Each cycle of the SBRs was 48 h, containing 43 h aeration, 4 h settling, 0.5 h decant and 0.5 h fill. In each cycle, half of the sewage was discharged manually and equal volume of fresh medium was replenished, thus the final indole concentration was 200 mg/L. All SBRs were operated under identical conditions.

### DNA extraction and pyrosequencing

Original activated sludge and sludge samples at the final cycle of each stage were collected and stored at −80 °C before use. The genomic DNA was extracted using PowerSoil® DNA Isolation Kit (MO BIO laboratories, CA USA). Concentration of DNA was determined by Nano drop. Primers 515F (5′-GTG CCA GCM GCC GCG GTA A-3′) and 806R (5′-GGA CTA CHV GGG TWT CTA AT-3′) with different barcodes for the V4 region of 16S rRNA gene were used for sequencing^48^. DNA library was constructed and run on the Miseq Illumina.

### Pyrosequencing result analysis

The raw sequences were joined and treated as previous described^24^. Operational taxonomic units (OTUs) were generated by UPARSE program. The taxonomic assignment was conducted using RDP classifier with a confidence cutoff of 0.5. The figures were drawn using SigmaPlot 11.0 and Excel. For statistical analysis, the data for each group were the average of its quadruplicate values.

### Strain isolation, identification and degradation analysis

Indole-degrading strains were isolated from the activated sludge samples of each stage. Isolation medium consisted of (g/L) 2.0 (NH_4_)_2_SO_4_, 2.0 KH_2_PO_4_, 3.28 Na_2_HPO_4_·12H_2_O and 0.00025 FeCl_3_ was used for strain screening. The activated sludges from each stage were utilized to screen pure cultures using serial dilution method. Finally, four stains were obtained which could use indole as the sole carbon and energy source. 16S rRNA genes of the isolated strains were amplified using universal primers and sequenced in Sangon Biotech Co. Ltd. (Shanghai, China). The sequences were aligned by Clustal X 1.8 and phylogenetic tree was obtained by MEGA 5.0. The strain growth and indole degradation experiments were conducted in 100 mL reaction systems under the condition of 30 °C and 150 r/min. The degradation medium consisted of (g/L) 0.1 MgSO_4_, 2.0 KH_2_PO_4_, 3.28 Na_2_HPO_4_·12H_2_O and 0.00025 FeCl_3,_ which did not contain extra nitrogen source. The original inoculum size was 5% and samples were taken at certain intervals for strain growth and indole concentration determination.

### Analytical methods

Indole concentration was measured by HPLC analysis (Hitachi Primaide, Japan)^27^. Strain growth was characterized by optical density at 660 nm (OD_660_) using UV-vis spectrophotometer (Metash UV-9000, China). The indole transformation products at the final cycle of each stage were analyzed using HPLC-MS analysis on a quadrupole ion trap instrument (Agilent HP 1100 LC/MSD, USA) equipped with an atmospheric pressure chemical ionization (APCI) source.

## Additional Information

**Accession codes**: The 16S rRNA gene sequences of IDO1, IDO2, IDO3 and IDO4 were deposited in GenBank under accession number of KP895478, KP895479, KP895480 and KP895481 respectively. The raw sequencing data of the present study have been submitted to NCBI Sequence Read Archive (http://www.ncbi.nlm.nih.gov/sra/) with the project accession number of SRP2052567.

**How to cite this article**: Ma, Q. *et al.* Systematic investigation and microbial community profile of indole degradation processes in two aerobic activated sludge systems. *Sci. Rep.* **5**, 17674; doi: 10.1038/srep17674 (2015).

## Supplementary Material

Supplementary Information

## Figures and Tables

**Table 1:** Sequence numbers, richness and alpha diversity of the sludge samples.

Index	System A				System B			
	Original	Stage I	Stage II	Stage III	Original	Stage I	Stage II	Stage III
Sequences before resample	18077	26440	35566	34785	18316	25890	36632	22891
Chao1	800 ± 56	412 ± 10	324 ± 117	213 ± 44	461 ± 4	529 ± 63	274 ± 51	183 ± 17
OTU number	629 ± 8	297 ± 9	179 ± 24	128 ± 28	372 ± 8	373 ± 29	188 ± 40	108 ± 14
Shannon index	5.10 ± 0.02	3.18 ± 0.17	2.54 ± 0.41	2.40 ± 0.41	3.47 ± 0.01	3.96 ± 0.22	2.35 ± 0.38	1.73 ± 0.18
Simpson index	0.01 ± 0	0.16 ± 0.05	0.16 ± 0.07	0.18 ± 0.07	0.14 ± 0	0.05 ± 0.01	0.20 ± 0.06	0.31 ± 0.03

Chao1 richness estimator: a higher number indicates higher richness. Shannon index (H): a higher value represents more diversity. Simpson Index (D): a higher value represents less diversity.

**Table 2:** Significance tests of the microbial communities based on Jaccard distances.

Groups	MRPP	ANOSIM	Adonis
	*P*	*P*	*P*
A Stage I vs. A Stage II	0.034	0.022	0.001
A Stage I vs. A Stage III	0.028	0.031	0.01
A Stage II vs. A Stage III	0.035	0.031	0.001
B Stage I vs. B Stage II	0.026	0.031	0.003
B Stage I vs. B Stage III	0.026	0.031	0.001
B Stage II vs. B Stage III	0.03	0.023	0.001
A Stage I vs. B Stage I	0.025	0.025	0.001
A Stage II vs. B Stage II	0.027	0.037	0.001
A Stage III vs. B Stage III	0.023	0.036	0.001

Adonis, permutational multivariate analysis of variance with the Adonis function. ANOSIM, analysis of similarity. MRPP, multiresponse permutation procedure. *P* value less than 0.05 means significant.
