# Supplementary material for: Systematic investigation and microbial community profile of indole degradation processes in two aerobic activated sludge systems
Source: Sci Rep. 2015 Dec 11;5:17674. doi: 10.1038/srep17674 (PMC4675989; doi:10.1038/srep17674)
Supplement: Supplementary Information [file srep17674-s1.doc]

***Supporting Information* for *Scientific Reports***

**Systematic investigation and microbial community profile of indole degradation processes in two aerobic activated sludge systems**

Qiao Ma, Yuanyuan Qu, Xuwang Zhang, Ziyan Liu, Huijie Li, Zhaojing Zhang, Jingwei Wang, Wenli Shen & Jiti Zhou

Key Laboratory of Industrial Ecology and Environmental Engineering (Ministry of Education), School of Environmental Science and Technology, Dalian University of Technology, Dalian 116024, People’s Republic of China

Address correspondence to, Yuanyuan Qu, School of Environmental Science and Technology, Dalian University of Technology, Dalian 116024, PR China. Tel, +86-411-84706250; E-mail, [qyy@dlut.edu.cn](mailto:qyy@dlut.edu.cn)

**Table S1**

Isolation of indole-degrading strains from the bioreactors.

| **Strain** | **Stage I** | | **Stage II** | | **Stage III** | |
| --- | --- | --- | --- | --- | --- | --- |
|  | A | B | A | B | A | B |
| ***Comamonas* sp. IDO1** | + | + | + | + | + | - |
| ***Comamonas* sp. IDO2** | + | - | - | - | - | - |
| ***Burkholderia* sp. IDO3** | - | - | + | + | + | + |
| ***Xenophilus* sp. IDO4** | - | - | - | - | + | - |

‘+’ means that strain was obtained at this stage, and ‘-’ means that strain was not obtained at this stage.


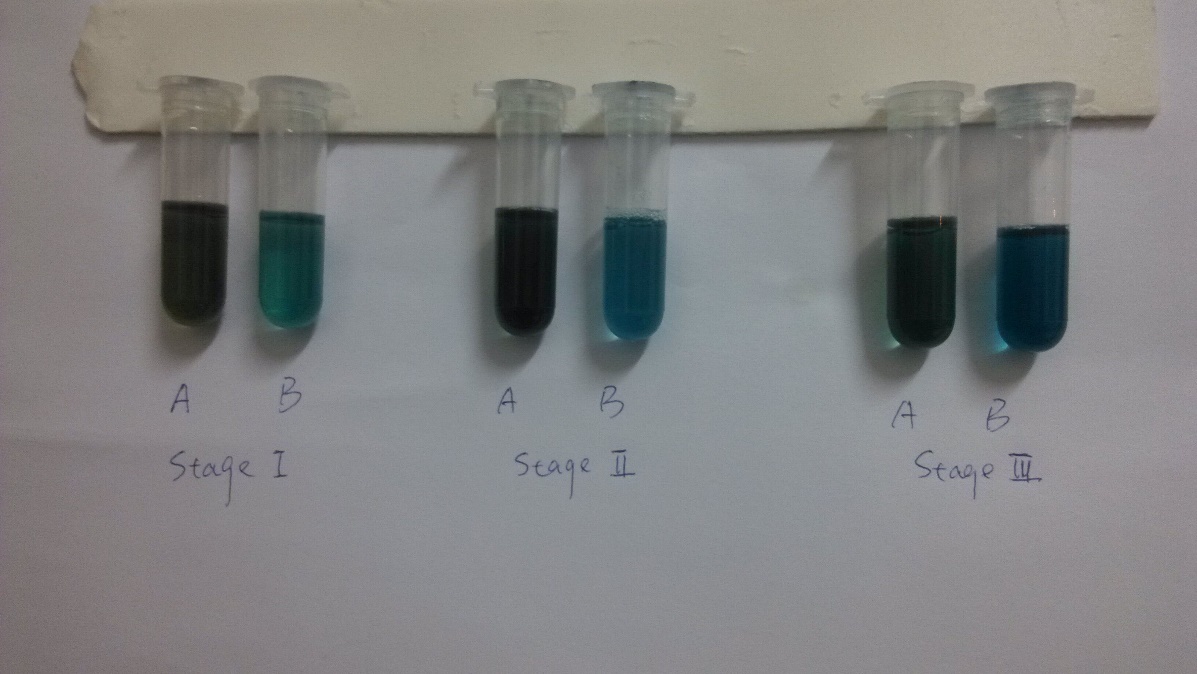


**Figure S1. Transformation products dissolved in dimethyl sulfoxide**. Samples were taken at the end of each stage and centrifuged. The precipitation was dissolved with dimethyl sulfoxide. From left to right, system A stage I, system B stage I, system A stage II, system B stage II, system A stage III and system B stage III.


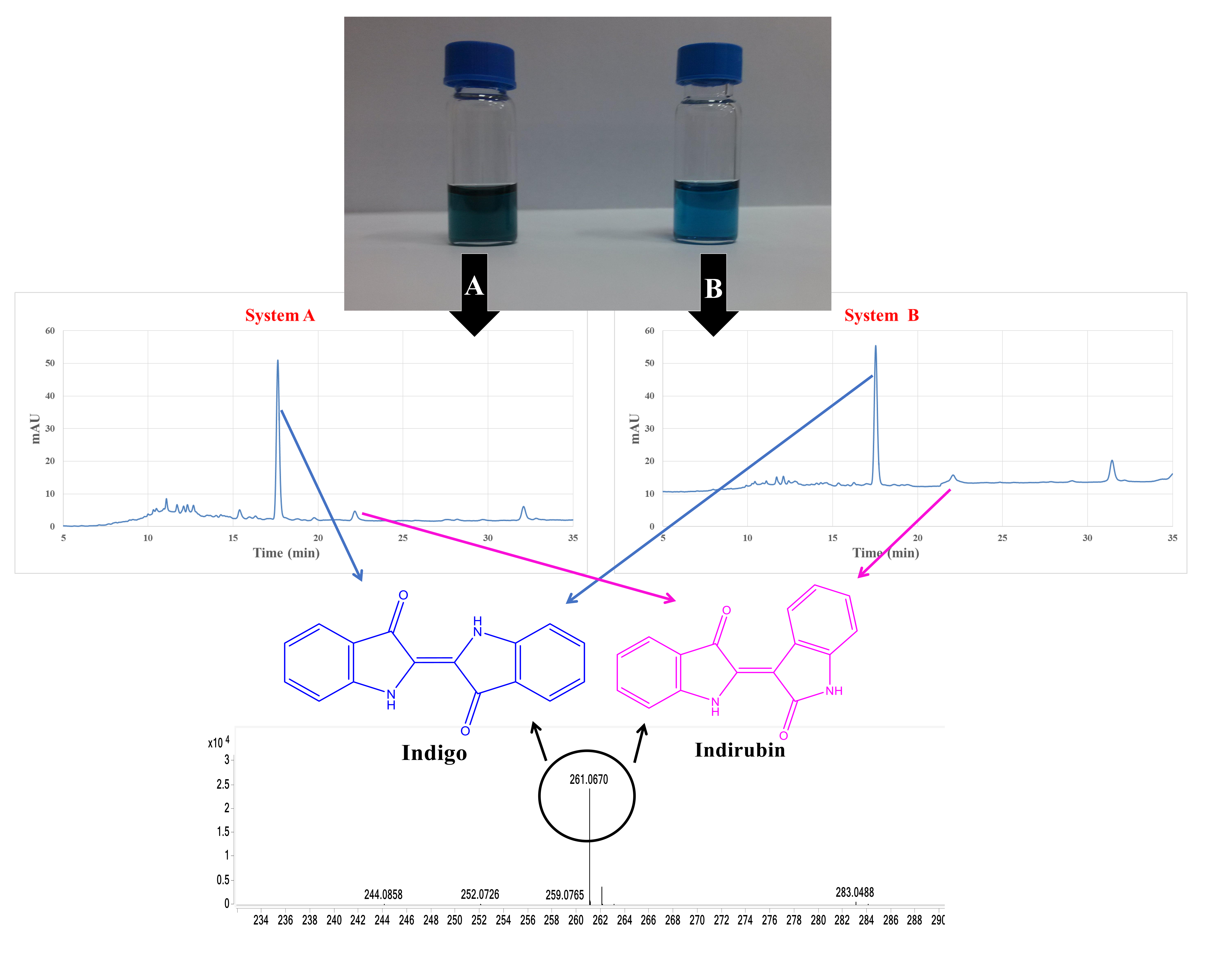


**Figure S2. Identification of the products in sludge**. Sludge samples were the taken from the stage III of both systems. Retention time of indigo and indirubin were compared with standards and further identified by HPLC-MS.





**Figure S3. Heat map of all samples at OTU level.** Cluster 3.0 is used to treat data and figure is visualized by Treeview.

**A**


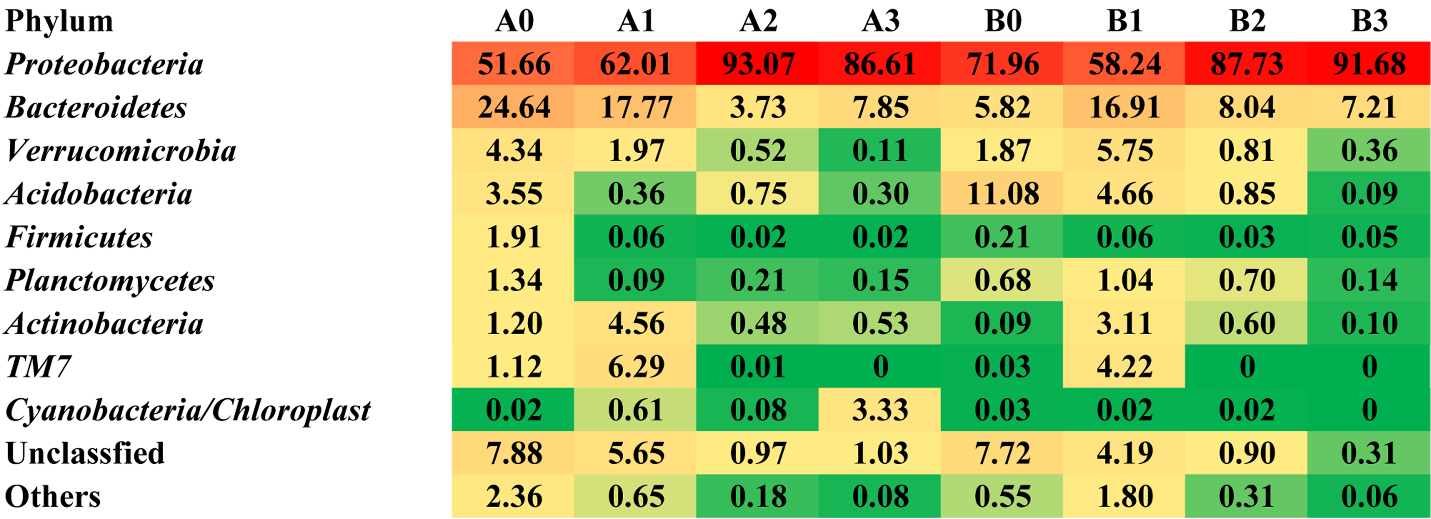


**B**


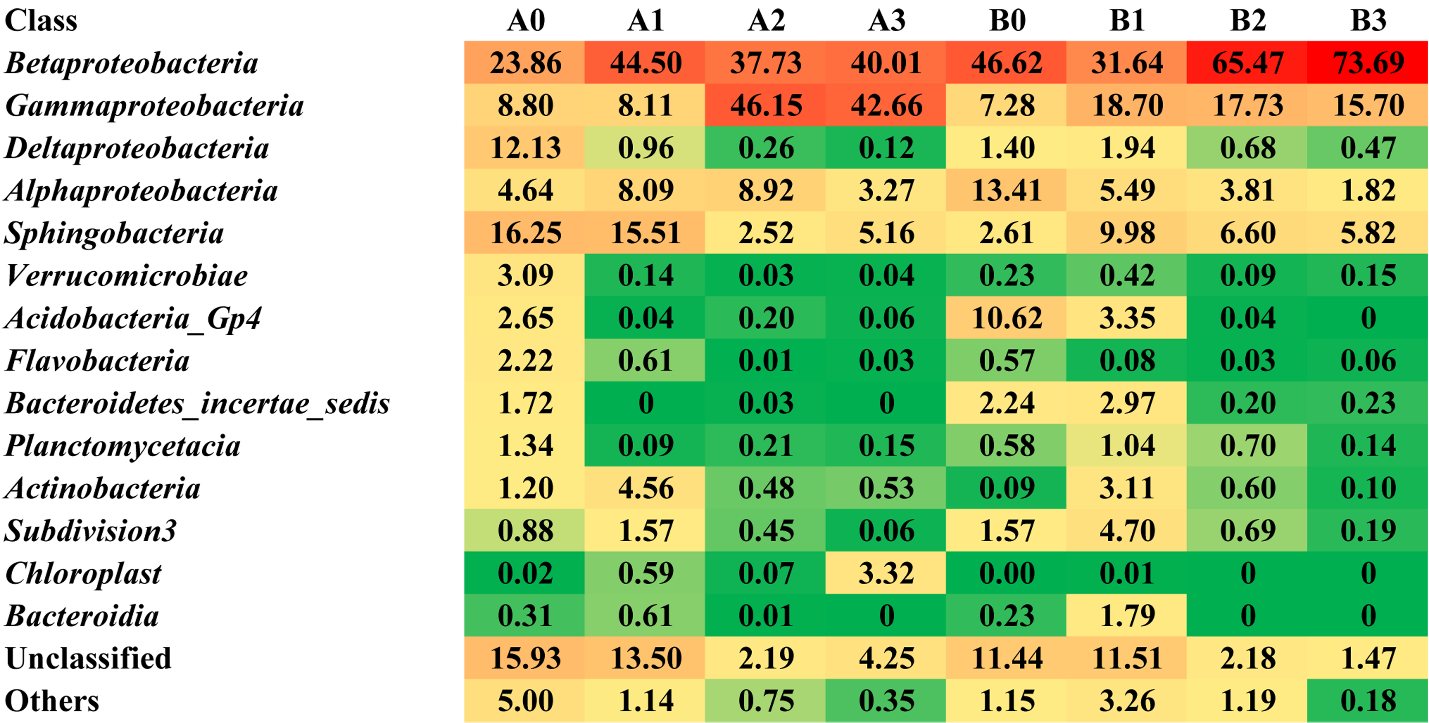


**Figure S4. Major phyla (A) and classes (B) of all groups.** Major means average sequence percentage is above 1% in any group.
